# Isolated Congenital Ossicular Chain Anomalies

**DOI:** 10.7759/cureus.112671

**Published:** 2026-07-14

**Authors:** Natsuko Obara, Hiroshi Okuda, Noriko Nagase, Ryoukichi Ikeda, Takenori Ogawa

**Affiliations:** 1 Otolaryngology-Head and Neck Surgery, Gifu University Graduate School of Medicine, Gifu, JPN

**Keywords:** conductive hearing loss, congenital middle ear anomaly, incudostapedial discontinuity, incus long process defect, ossicular malformation, ossiculoplasty, siblings

## Abstract

Isolated congenital ossicular chain anomalies (COCAs) are an uncommon cause of conductive hearing loss in patients with a normal external auditory canal and tympanic membrane, and familial clustering is rarely described. We report three siblings with non-progressive conductive hearing loss and normal otoscopic findings. Two siblings exhibited bilateral involvement, whereas the third had unilateral disease with a normal contralateral ear. High-resolution temporal bone computed tomography suggested incudostapedial (I-S) discontinuity caused by defects in the long process of the incus, without external or inner ear anomalies. Exploratory tympanotomy in two siblings confirmed congenital-appearing I-S discontinuity without evidence of chronic otitis media. Ossiculoplasty using autologous incus interposition (type III reconstruction) achieved marked postoperative improvement. There was no reported family history of middle ear malformation outside this sibship, and a targeted next-generation sequencing panel (63 known deafness genes) performed in two siblings did not identify a definitive pathogenic variant. This case series highlights that COCAs can cluster among siblings and may show variable laterality (bilateral and unilateral) within the same family, underscoring the value of early imaging and surgical evaluation when conductive hearing loss persists despite normal tympanic membranes.

## Introduction

Isolated congenital ossicular chain anomalies (COCAs) are an uncommon cause of conductive hearing loss in patients with a normal external auditory canal and an intact tympanic membrane [1]. In children, persistent conductive hearing loss is frequently attributed to otitis media with effusion [2]; consequently, COCAs may be underrecognized, particularly when newborn hearing screening is not performed or when the disease is unilateral. COCAs have been classified according to intraoperative findings. The Teunissen and Cremers classification remains widely referenced and categorizes congenital anomalies based primarily on the status of the stapes footplate and the oval and round windows, which is useful for surgical decision-making and prognostication [3]. Funasaka classified congenital ossicular anomalies without external ear anomalies as (1) fixation of the malleus and/or incus, (2) disconnection of the incudostapedial joint, and (3) fixation of the stapes [4].

Among COCA subtypes, incudostapedial (I-S) discontinuity due to absence or hypoplasia of the long process of the incus is a characteristic phenotype [5,6]. Distinguishing congenital I-S discontinuity from acquired ossicular erosion (e.g., caused by chronic otitis media or congenital cholesteatoma) is clinically important because both may present with conductive hearing loss and a seemingly normal tympanic membrane; however, congenital cases generally lack a history of inflammation and do not show middle ear soft-tissue changes on computed tomography (CT) [5]. Surgical outcomes are often favorable when the stapes footplate is mobile and reconstruction is feasible [7,8].

Most COCA cases are sporadic. Familial reports exist but are limited, including multigenerational pedigrees, mother-daughter pairs, and twin cases, suggesting a hereditary contribution in selected families [9-13].

Prior reports have exclusively involved female patients and have largely described bilateral disease. Here, we describe three siblings with congenital ossicular chain anomalies consistent with incudostapedial discontinuity due to defects of the long process of the incus, including two bilateral cases and one unilateral case with a normal contralateral ear, thereby extending the previously reported phenotypic spectrum to include both a male case and unilateral involvement.

## Case presentation

Family overview

Three siblings born to non-consanguineous parents were evaluated for congenital, non-progressive conductive hearing loss. None had a history of recurrent acute otitis media, chronic otorrhea, ear trauma, or previous ear surgery. Otoscopic examination showed normal auricles, normal external auditory canals, and intact tympanic membranes without retraction pockets, effusion, or scarring. No craniofacial anomalies, developmental delay, or systemic findings suggestive of syndromic disease were identified. The parents reported no family history of middle ear malformation among first- or second-degree relatives other than these three siblings.

Case 1

**Figure 1:**
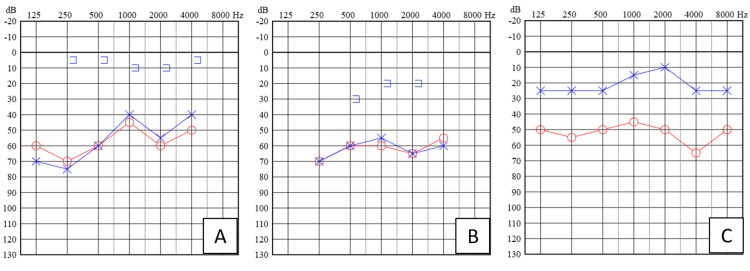
Preoperative pure-tone audiograms for Case 1 (A), Case 2 (B), and Case 3 (C). (A) Case 1 shows bilateral moderate hearing loss. A substantial air-bone gap is documented on the left side, whereas reliable right bone-conduction thresholds cannot be obtained because of test limitations. (B) Case 2 shows bilateral moderate hearing loss. A large air-bone gap is documented on the left side, whereas right bone-conduction thresholds are difficult to measure reliably at that age. (C) Case 3 shows unilateral right-sided conductive hearing loss with normal hearing in the left ear.

Case 1 (female) was first evaluated at the age of four years after she complained of difficulty hearing. Newborn hearing screening had not been performed. Speech and language development were acceptable by parental report, but hearing difficulty became evident in daily conversation. Pure-tone audiometry revealed moderate hearing loss with a four-frequency PTA of 52.5 dB HL (right) and 48.8 dB HL (left) (Figure 1A).

**Figure 2:**
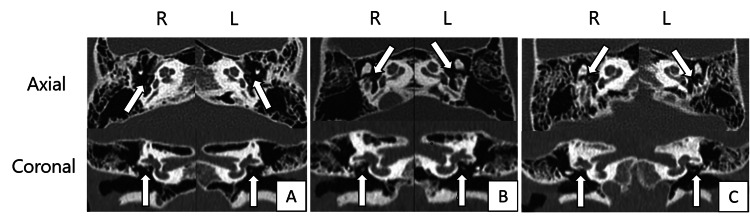
Temporal bone computed tomography images for Case 1 (A), Case 2 (B), and Case 3 (C). Axial images are shown in the upper panels, and coronal images are shown in the lower panels. (A) Case 1 shows bilateral absence or hypoplasia of the long process of the incus, suggesting bilateral incudostapedial discontinuity. (B) Case 2 shows bilateral defects of the long process of the incus, consistent with bilateral incudostapedial discontinuity. (C) Case 3 shows a right-sided defect or hypoplasia of the long process of the incus, whereas the left ossicular chain is morphologically normal. White arrows indicate the affected long process of the incus.

A substantial air-bone gap (ABG) was documented on the left, whereas reliable right bone-conduction thresholds could not be obtained due to test limitations. Tympanometry showed type Ad curves bilaterally. Computed tomography (CT) demonstrated bilateral defects or hypoplasia of the long process of the incus with suspected I-S discontinuity (Figure 2A).

**Figure 3:**
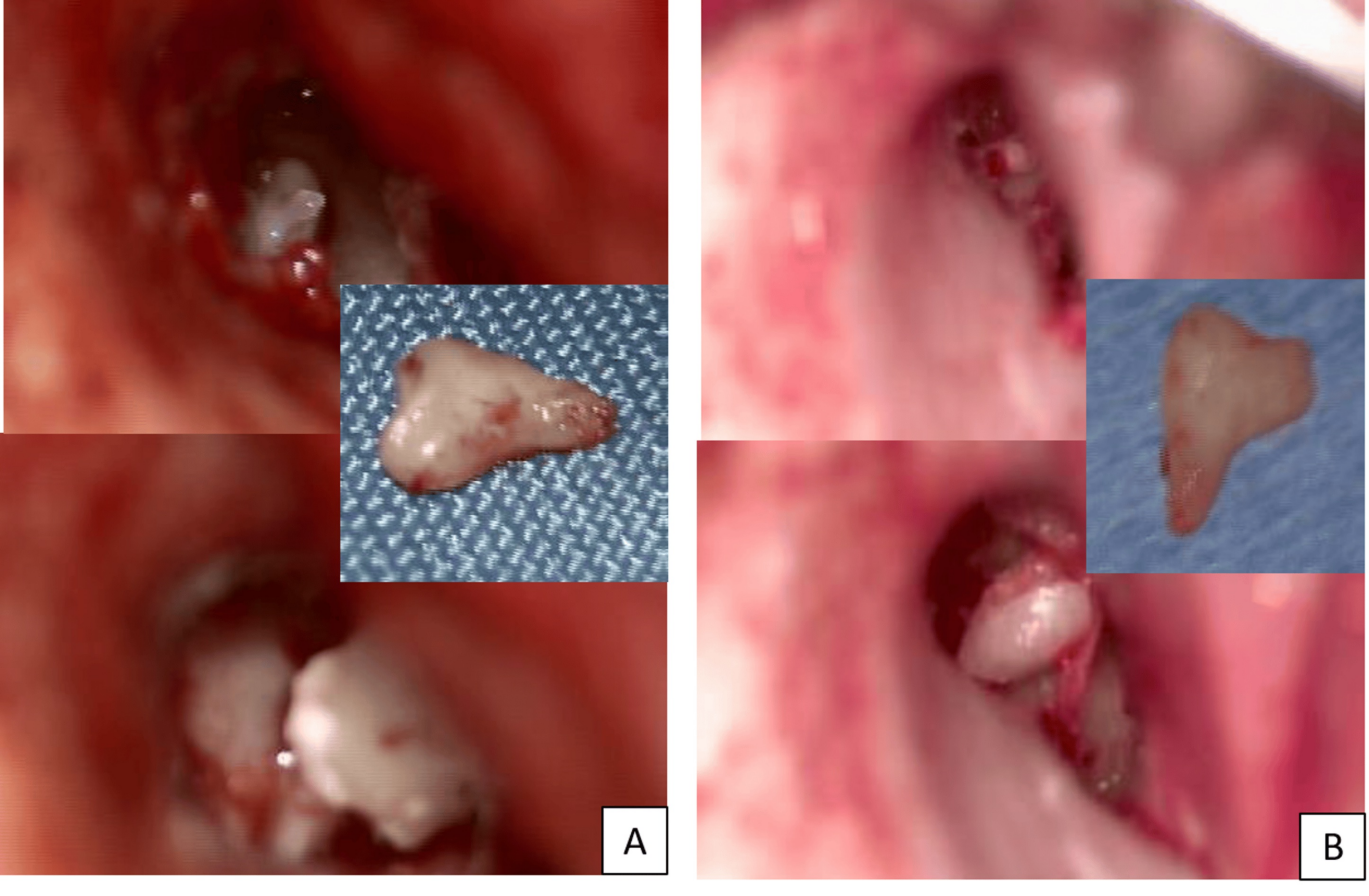
Intraoperative findings in Case 1 and Case 2. (A) Case 1, right ear. The upper panel shows the stapes region and demonstrates incudostapedial discontinuity caused by the absence of the long process of the incus. The middle panel shows the removed incus, confirming a defect of the long process. The lower panel shows the reconstructed ossicular chain after autologous incus interposition. (B) Case 2, right ear. The upper panel shows incudostapedial discontinuity around the stapes. The middle panel shows the incus with a defective long process. The lower panel shows the postoperative appearance after ossicular chain reconstruction using autologous incus interposition.

No CT evidence of chronic otitis media was present. At the age of five years, right tympanoplasty revealed a congenitally appearing I-S discontinuity due to the absence or hypoplasia of the long process of the incus. The stapes superstructure was present, and footplate mobility was confirmed. No granulation tissue, cholesteatoma, or inflammatory adhesions were seen. Ossiculoplasty was performed using incus interposition (type IIIi reconstruction) (Figure 3A).

**Figure 4:**
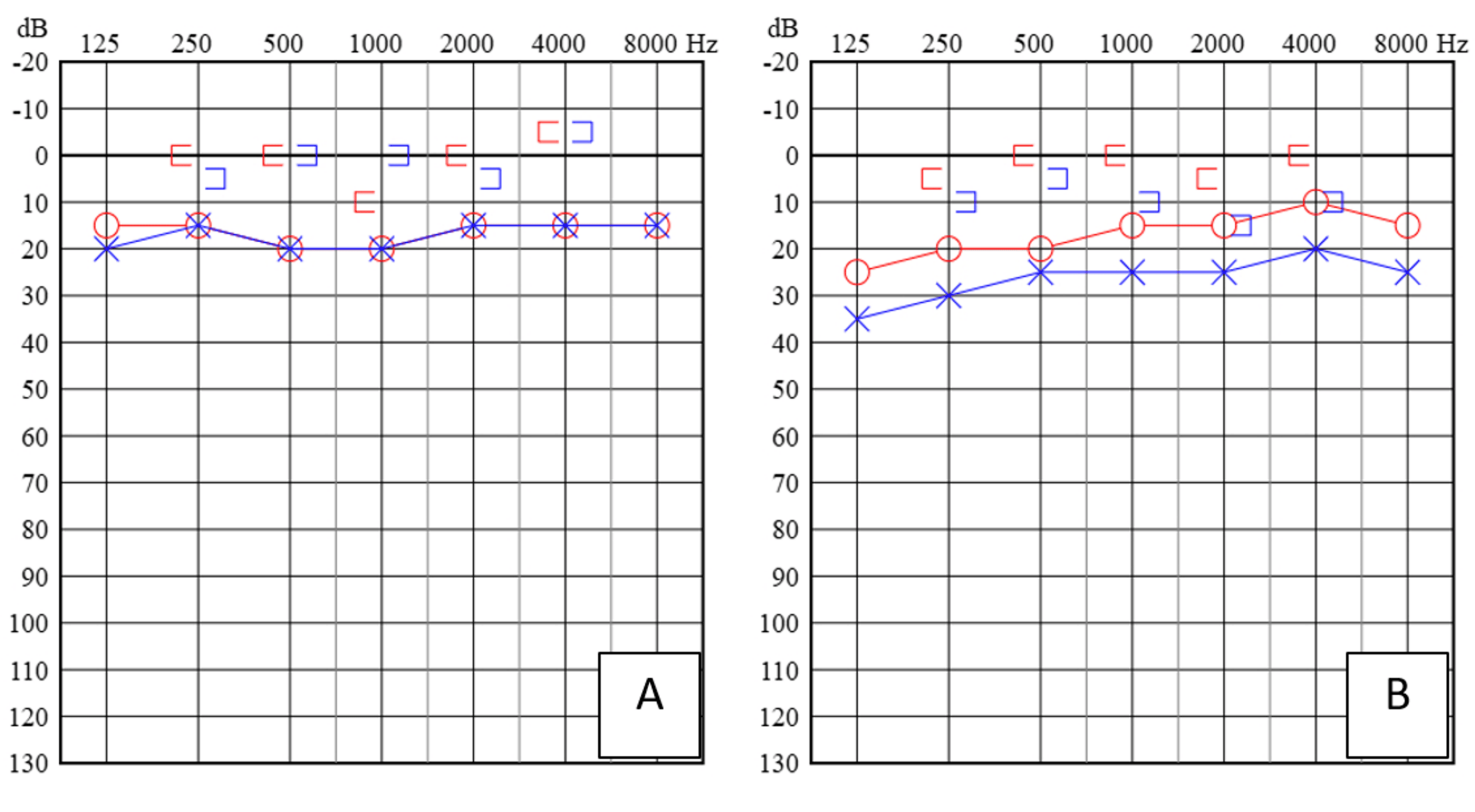
Postoperative pure-tone audiograms for Case 1 (A) and Case 2 (B). (A) Case 1 showed marked postoperative hearing improvement in both ears after bilateral ossiculoplasty, with air-bone gap closure to within 20 dB. (B) Case 2 showed postoperative hearing improvement in both ears after bilateral ossiculoplasty, with air-bone gap closure to within 20 dB.

At the age of seven years, left tympanoplasty showed similar findings, and the same reconstruction was performed. At the age of nine years three months, postoperative PTA improved to 18.8 dB HL bilaterally, with ABG closure to within 20 dB (Figure 4A). No postoperative vertigo, facial nerve dysfunction, or sensorineural deterioration was observed.

Case 2

Case 2 was the younger brother of Case 1. He was identified by newborn hearing screening, which showed bilateral "refer" results. Auditory brainstem response testing at a local clinic suggested bilateral moderate hearing loss, and early intervention, including amplification, was initiated. At five months of age, auditory steady-state response findings were consistent with bilateral moderate hearing loss.

At two years of age, behavioral audiometry showed pure-tone average (PTA) values of 61.3 dB HL in the right ear and 58.8 dB HL in the left ear (Figure 1B). A large air-bone gap was documented on the left side, whereas right bone-conduction thresholds were difficult to measure reliably at that age. Temporal bone CT at two years of age demonstrated bilateral defects of the long process of the incus, consistent with incudostapedial discontinuity, without external or inner ear anomalies or inflammatory middle ear changes (Figure 2B).

At four years of age, right exploratory tympanotomy confirmed incudostapedial discontinuity due to a defect of the long process of the incus with a mobile stapes footplate. Type III reconstruction with autologous incus interposition was performed (Figure 3B). At six years of age, left surgery revealed a similar defect. The stapes superstructure was somewhat globular, but the footplate remained mobile. The same reconstructive strategy was applied.

At six years and four months of age, the postoperative PTA improved to 16.3 dB HL in the right ear and 25.0 dB HL in the left ear, with air-bone gap closure to within 20 dB bilaterally (Figure 4B).

Case 3

Case 3 was the youngest sibling, a female, who was referred after newborn hearing screening showed a right-sided "refer" result. Auditory brainstem response testing at five months of age demonstrated a right threshold of 55 dB nHL with identifiable wave V and a left threshold of 20 dB nHL, suggesting unilateral hearing impairment with normal contralateral hearing.

At two years of age, conditioned orientation response testing suggested thresholds of approximately 30 dB HL. At three years of age, simplified pure-tone testing showed a threshold of 47.5 dB HL in the right ear and 16.3 dB HL in the left ear, consistent with unilateral conductive hearing loss (Figure 1C). Speech and language development were age-appropriate, and no additional educational support was required.

Temporal bone CT at two years of age showed a right-sided defect or hypoplasia of the long process of the incus, consistent with incudostapedial discontinuity. The left ossicular chain was morphologically normal (Figure 2C). She remains under follow-up. Given the favorable outcomes in her siblings and the stable clinical course, right ossiculoplasty is planned before school entry.

Genetic testing

Cases 1 and 2 underwent next-generation sequencing using a targeted panel of 63 known deafness-associated genes [14]. No definitive pathogenic variant explaining the phenotype was identified. This negative result does not exclude a genetic etiology because causative variants may lie outside the analyzed panel, involve non-coding or structural variants, or affect developmental pathways not typically captured by standard hearing-loss gene panels.

## Discussion

We report three siblings with COCAs manifesting as I-S discontinuity due to defects of the long process of the incus. Previous reports have predominantly described bilateral involvement in female patients, whereas in our series, two patients were female and one was male, and one sibling exhibited unilateral disease (Table 1). 

**Table 1:** Published familial reports of isolated ossicular malformations and the present case.

Study	Family structure	Sex	Laterality	Main lesion(s)
Higashi et al. (1987) [9]	Two generations	Female	Bilateral	Incus long process defect; fibrous connection at I–S joint
Wehrs (1999) [6]	Three generations	Female	Bilateral	Incus long process defect; stapes suprastructure defect
Lee et al. (2009) [10]	Three generations	Female	Bilateral	Incus long process defect; stapes fixation
Nakanishi et al. (2011) [11]	Two generations	Female	Bilateral	Incus long process defect; stapes fixation
Kidowaki et al. (2014) [12]	Identical twins	Female	Bilateral	Incus long process defect
Matsunobu et al. (2025) [13]	Two generations	Female	Bilateral	Incus long process defect; stapes fixation in one case
	(mother + 2 maternal aunts + daughte)			
Present cases	Three siblings	Female ×2, Male ×1	Bilateral ×2 Unilateral ×1	Incus long process defect; partial stapes anomaly in one ear

These findings suggest for the first time that familial congenital middle ear anomalies can occur in males and may present unilaterally, expanding the phenotypic spectrum of familial ossicular malformations.

Congenital versus acquired mechanisms

Absence or hypoplasia of the long process of the incus has been discussed as either a congenital anomaly or the result of inflammatory resorption. Park et al. noted that although a congenital origin is likely in many cases, particularly when associated with other congenital morphology, an inflammatory component cannot be completely excluded in selected patients [5]. Another possible acquired mechanism is destruction of the incudostapedial joint by a preexisting cholesteatoma, followed by spontaneous regression or evacuation of the lesion. Because cholesteatoma can erode the ossicular chain, especially the long process of the incus and the stapes superstructure, this possibility should be considered when incudostapedial discontinuity is encountered. However, in our series, there was no history of otorrhea or recurrent middle ear infection, the tympanic membranes were normal without retraction pockets, keratin debris, scarring, or adhesive changes, and CT showed no evidence of chronic otitis media, residual soft-tissue lesions, mastoid opacification, or other acquired destructive changes. In addition, the familial clustering among siblings and the congenital-appearing intraoperative morphology strongly supported a congenital etiology. Although a previously resolved cholesteatoma cannot be completely excluded, we consider it unlikely in the present cases.

Classification and surgical prognosis

Our operated ears were consistent with anomalies associated with a mobile stapes footplate, for which surgical results are generally favorable. In a series of congenital ossicular chain anomalies with a mobile footplate, postoperative ABG closure was achieved in a substantial proportion of ears [7]. A prospective pediatric series also reported favorable outcomes for congenital ossicular chain malformations with a mobile stapes, supporting surgical reconstruction in appropriately selected children [8]. Consistent with these reports, the operated ears in Cases 1 and 2 achieved ABG closure to within 20 dB.

Familial reports and variable laterality

Familial clustering of COCAs remains rare but has been reported in multigenerational families and mother-daughter pairs [9-11]. Twin cases further support a developmental and possibly hereditary contribution in selected families [12]. Recently, Matsunobu et al. reported a familial congenital ossicular anomaly involving four female relatives across two generations [13]. All affected members shared I-S discontinuity associated with absence or defect of the long process ("long limb") of the incus, and one additionally had stapes fixation. This report strengthens the evidence that incus long process defects with I-S discontinuity can recur within families. Our family differs in that clustering was confined to siblings with unaffected parents by history, and laterality varied: two siblings had bilateral disease, whereas one had unilateral disease with a normal contralateral ear. This pattern may reflect autosomal recessive inheritance, reduced penetrance, variable expressivity, or parental germline mosaicism. The negative targeted deafness gene panel does not exclude a genetic contribution because causative variants may involve genes not included in standard panels or noncoding/structural variants. From a counseling perspective, our findings suggest that even unilateral conductive hearing loss with a normal tympanic membrane should prompt consideration of CCOA when a sibling is affected, and proactive evaluation of siblings may be warranted.

Clinical implications

This series illustrates the importance of integrating newborn hearing screening results, careful otoscopy, and CT findings when evaluating conductive hearing loss. Case 1 was not evaluated until she became symptomatic, whereas Cases 2 and 3 were detected early through screening, enabling earlier intervention and appropriate planning for surgical management. Because ossiculoplasty can provide substantial functional improvement in selected ears, early recognition of CCOA can reduce the duration of unnecessary medical therapy for presumed otitis media and facilitate timely rehabilitation.

## Conclusions

Three siblings demonstrated isolated congenital ossicular malformations consistent with incudostapedial discontinuity caused by defects of the long process of the incus. Two siblings had bilateral involvement and achieved excellent postoperative hearing improvement after ossiculoplasty, whereas one sibling had unilateral involvement with a normal contralateral ear. The clustering of cases within this sibship, together with variable laterality, supports the possibility of a hereditary contribution. Congenital ossicular anomalies should be included in the differential diagnosis of congenital conductive hearing loss with normal tympanic membranes, and early CT and surgical evaluation should be considered when clinically appropriate.
